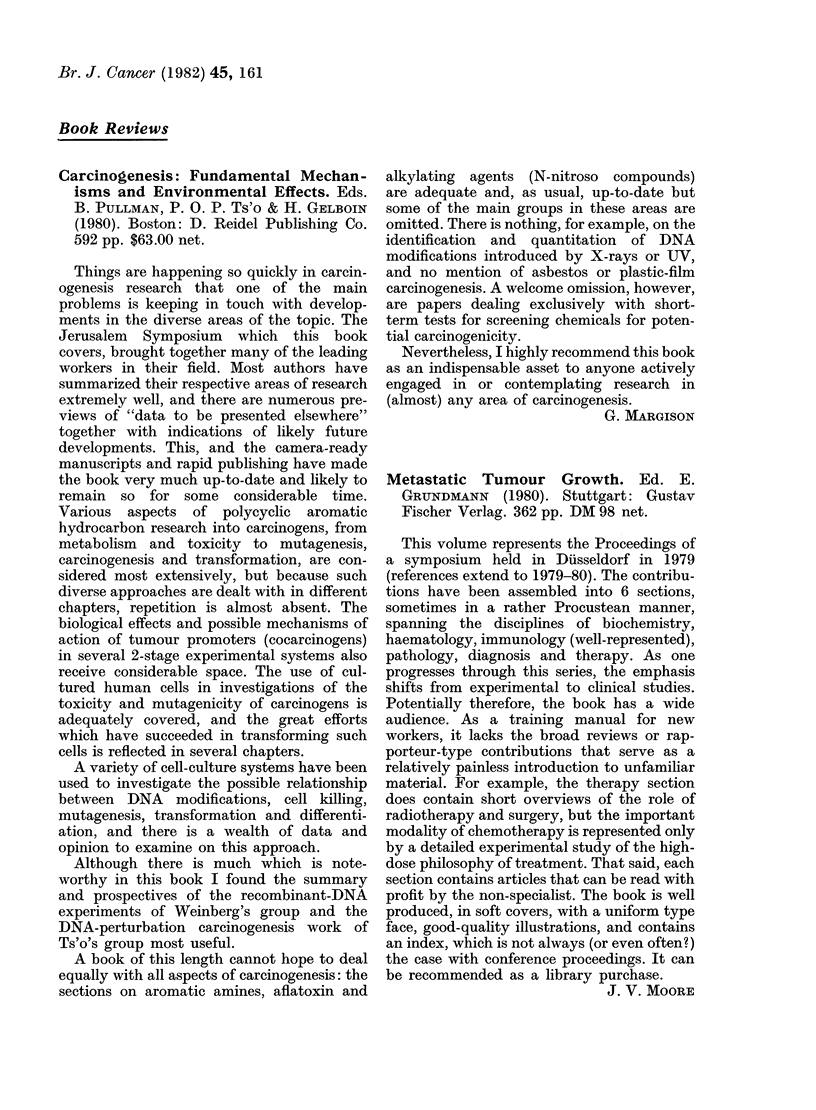# Metastatic Tumour Growth

**Published:** 1982-01

**Authors:** J. V. Moore


**Metastatic Tumour Growth.** Ed. E. GRUNDMANN (1980). Stuttgart: Gustav Fischer Verlag. 362 pp. DM 98 net.

This volume represents the Proceedings of a symposium held in Düsseldorf in 1979 (references extend to 1979–80). The contributions have been assembled into 6 sections, sometimes in a rather Procustean manner, spanning the disciplines of biochemistry, haematology, immunology (well-represented), pathology, diagnosis and therapy. As one progresses through this series, the emphasis shifts from experimental to clinical studies. Potentially therefore, the book has a wide audience. As a training manual for new workers, it lacks the broad reviews or rapporteur-type contributions that serve as a relatively painless introduction to unfamiliar material. For example, the therapy section does contain short overviews of the role of radiotherapy and surgery, but the important modality of chemotherapy is represented only by a detailed experimental study of the high-dose philosophy of treatment. That said, each section contains articles that can be read with profit by the non-specialist. The book is well produced, in soft covers, with a uniform type face, good-quality illustrations, and contains an index, which is not always (or even often?) the case with conference proceedings. It can be recommended as a library purchase.

J. V. MOORE